# Investigating interassay variability between direct oral anticoagulant calibrated anti–factor Xa assays: a substudy of the perioperative anticoagulation use for surgery evaluation (PAUSE) trial

**DOI:** 10.1016/j.rpth.2025.102899

**Published:** 2025-05-23

**Authors:** Ryan M. Baker, Rita Selby, Karen A. Moffat, Melanie St John, Alex C. Spyropoulos, Sam Schulman, James Douketis

**Affiliations:** 1 Temerty Faculty of Medicine, University of Toronto, Ontario, Canada; 2 Department of Laboratory Medicine and Pathobiology, University of Toronto, Ontario, Canada; 3 Department of Medicine, University of Toronto, Ontario, Canada; 4 Hamilton Regional Laboratory Medicine Program, Ontario, Canada; 5 Department of Medicine, McMaster University, Ontario, Canada; 6 The Donald and Barbara Zucker School of Medicine at Hofstra/Northwell, Hempstead, New York, USA; 7 Institute of Health System Science, The Feinstein Institutes for Medical Research, Manhasset, New York, USA; 8 Anticoagulation and Clinical Thrombosis Services, Northwell Health at Lenox Hill Hospital, New York, New York, USA; 9 Thrombosis and Atherosclerosis Research Institute, McMaster University, Ontario, Canada

**Keywords:** apixaban, blood coagulation tests, clinical laboratory techniques, factor Xa inhibitors, rivaroxaban

## Abstract

**Background:**

Direct oral anticoagulant calibrated anti-factor Xa (FXa) assays can assess residual anticoagulant levels in patients requiring urgent procedures or surgery. However, previous studies have shown variability between anti-FXa levels determined by different instrument-reagent combinations. This may be related to use of lyophilized samples, direct oral anticoagulant-spiked plasma, or interlaboratory variation.

**Objectives:**

1) Determine the interassay variability in anti-FXa levels using 3 common instrument-reagent combinations. 2) Determine if differences between these combinations are clinically relevant.

**Methods:**

Seventy apixaban and 59 rivaroxaban samples from participants in the Perioperative Anticoagulation Use for Surgery Evaluation trial were simultaneously tested using Biophen reagents on the BCS XP analyzer (Siemens), HemosIL reagents on the ACL TOP analyzer (Werfen), and Stago reagents on the STA CompactMAX analyzer (Diagnostica Stago). Interassay correlations were analyzed at the predetermined cutoff of 30 ng/mL and compared with median anti-FXa levels.

**Results:**

Anti-FXa levels showed moderate-to-very strong correlations for apixaban (*r* = 0.7271-0.9467) and rivaroxaban (*r* = 0.6531-0.9702). Anti-FXa levels were also significantly different between all instrument-reagent combinations in the < 30 ng/mL group. In the ≥ 30 ng/mL group, apixaban was significantly different in all combinations, while rivaroxaban only differed between Biophen/BCS XP and Stago/STA CompactMAX. 7.8% (10/129) of samples were discrepantly classified across the 30 ng/mL threshold.

**Conclusions:**

Anti-FXa levels determined by 3 common instrument-reagent combinations show moderate-to-very strong correlations with each other. Although there are statistically significant differences between median anti-FXa levels these differences are not clinically significant, and result in discrepant classification across the 30 ng/mL threshold in only 7.8% of samples.

## Essentials

• DOAC levels may risk-stratify patients needing urgent procedures.
• We studied variability between DOAC levels using 3 common anti-FXa assays.
• Assays showed moderate-to-very strong correlations with each other.
• However, these differences were not clinically significant.

## 1 Background

Direct oral anticoagulants (DOACs) have largely replaced traditional anticoagulants as firstline agents due to their lack of routine monitoring, ease of use, and safety in the treatment and prevention of thromboembolic events [[1], [2], [3]]. Unlike anticoagulants such as warfarin and heparin, DOACs are effective at a fixed dose and do not require routine monitoring of DOAC-specific anti-factor Xa (FXa) levels for dose adjustment [4]. However, certain clinical scenarios, such as severe bleeding on a DOAC or a patient requiring DOAC interruption for urgent surgery, may require accurate measurement of DOAC levels, especially since routine coagulation studies like prothrombin time (PT) and activated partial thromboplastin time (aPTT) do not reliably determine the anticoagulant effect of DOACs [5,6]. Despite a lack of prospective studies correlating DOAC anti-FXa levels with clinical outcomes such as bleeding, a cutoff of 30 ng/mL for anti-FXa activity has been adopted for proceeding with surgery after DOAC interruption, based on pharmacodynamic studies and published subanalyses of clinical trials and retrospective studies [[7], [8], [9], [10]]. Data from recent external quality assessment (EQA) program surveys have raised concerns about significant interassay variability between the available DOAC anti-FXa assays on the same samples, at clinical decision points below 50 to 100 ng/mL [11,12]. The known limitation of EQA data is that survey samples consist of DOACs spiked into plasma and subsequently lyophilized to allow for mass production and transportation of the same sample between laboratories. It could be argued that these findings may not be generalizable to DOAC anti-FXa assays tested on fresh or fresh-frozen patient plasma samples in real-world settings. This is due to a theoretical concern that spiking DOACs into normal plasma samples does not account for metabolism, protein-binding, enzymatic reaction, or other factors that may impact either the levels of DOAC or their effects on an assay. Jennings et al. [13] showed that when comparing clinical samples to spiked samples, there were commutable results for rivaroxaban, low molecular weight heparin, and some factor (F)VIII concentrates, but not for dabigatran or unfractionated heparin [13]. Apixaban was not assessed though and as such, further research is needed to determine the extent of variability introduced by spiking samples in relation to apixaban. Additionally, a recent substudy of a larger prospective, multicenter registry study that compared 7 different chromogenic anti-FXa assays in patients on DOACs found variability occurring at both high and low DOAC concentrations depending on the assay used [14,15]. Interassay variability in anti-FXa assays has been previously reported [[16], [17], [18], [19], [20], [21], [22], [23]]. However, many of these studies used either spiked samples, lyophilized samples, or required samples to be transferred to different centers for analysis on the various analyzers.

The Perioperative Anticoagulation Use for Surgery Evaluation (PAUSE) cohort study evaluated 3007 patients with atrial fibrillation enrolled from 23 clinical centers across Canada, the United States, and Europe from 2014 to 2018 [24]. Participants were ≥ 18 years of age, were long-term users of apixaban, dabigatran, or rivaroxaban and were scheduled for an elective surgery or procedure. In addition to following a simple standardized perioperative DOAC therapy interruption and resumption strategy based on DOAC pharmacokinetic properties, procedure-associated bleeding risk, and creatinine clearance levels, all patients had a citrated plasma sample collected and stored centrally just before surgery for future analysis. This provided us an opportunity to address the aim of our study, which was to assess interassay variability in DOAC anti-FXa measurements at low concentrations while controlling for confounding factors. These factors were limited by using nonspiked, nonlyophilized frozen patient plasma samples analyzed simultaneously on 3 of the most commonly used instrument-reagent platforms at a single central laboratory, thus avoiding sample transport and repeated freeze-thaw cycles.

## 2 Methods

### 2.1 Study design

We conducted a substudy of the PAUSE trial, which was an international multicenter study that examined adult patients with atrial fibrillation who were on long-term therapy with a DOAC (either apixaban, rivaroxaban, or dabigatran) [24]. Our objectives were as follows: (1) to describe interassay variability in DOAC anti-FXa measurements using 3 of the most commonly used coagulation instrument-reagent platforms on the same citrated frozen plasma samples after prespecified DOAC interruption and (2) to determine if the differences between the different instrument-reagent combinations would have led to clinically relevant alternative decisions around proceeding with a procedure or surgery. In the PAUSE trial, patients had their DOAC treatment withheld prior to undergoing an elective surgery or procedure, either 1 day prior to a low-risk surgery or 2 days prior to a high-risk surgery. Blood was collected just before the procedure, and platelet poor sodium citrated plasma samples were stored at −70°C until analysis. Patients had previously consented to use of their plasma samples for potential future research studies. Baseline characteristics are shown in Table and DOAC dosing was at the discretion of the treating physician. From the 2624 patient plasma samples collected in the PAUSE study, we initially included 100 samples chosen at random and then increased our sample size at the clinically important threshold of 30 ng/mL by including all samples with sufficient remaining plasma volumes that were previously found to have anti-FXa levels between 30 and 50 ng/mL as determined during the initial PAUSE trial. The initial PAUSE trial utilized an instrument-reagent combination that included Hyphen BioMed reagents on a STAr-Evolution analyzer (Diagnostica Stago), which is a combination not used in our study to limit the introduction of a selection bias toward one of our combinations. Two patients in the apixaban group were not included in the analysis due to insufficient sample quantity after thawing. Six patients were excluded due to mislabeling, which resulted in the incorrect anti-FXa calibrator being used for their analysis. This led to a total sample size of 129 patients for this study.

Anti-FXa levels were determined simultaneously on the following 3 instrument-reagent combinations: STA reagents on the STA CompactMAX analyzer (Diagnostica Stago), HemosIL on the ACL TOP 300 analyzer (Werfen), and Biophen (Hyphen Biomed) on the BCS XP analyzer (Siemens). The following calibrators were used in this study: Biophen Apixaban Calibrator (Hyphen Biomed), Biophen Rivaroxaban Calibrator (Hyphen Biomed), HemosIL Apixaban Calibrator (Werfen), HemosIL Rivaroxaban Calibrator (Werfen), STA Apixaban Calibrator (Diagnostica Stago), STA Rivaroxaban Calibrator (Diagnostica Stago). International EQA program data and personal communication with Piet Meijer (External quality Control for diagnostic Assays and Tests (ECAT) Foundation) suggests that these are the commonest instrument-reagent platforms in current use to determine DOAC anti-FXa levels in clinical laboratories [25]. Testing was done at one central laboratory (Universal Biosensors’ Hemostasis Reference Laboratory) by 1 technologist, to eliminate interlaboratory and interoperator variability. Frozen samples were thawed in a 37 °C water bath for 5 minutes and mixed well prior to analysis. Anti-FXa assays were performed according to the manufacturer’s test protocol.

### 2.2 Statistical analysis

Results were analyzed using GraphPad Prism 9 (Mac OS). Correlation analysis for the anti-FXa levels determined by the 3 instrument-reagent combinations were done using the Spearman rank correlation coefficient with a 2-tailed *P* value of < .05 to determine statistical significance. Correlation strengths were based on values defined by Schober et al. [26]. Differences between instrument-reagent combinations and anti-FXa levels were determined using the paired Friedman test with Dunn multiple comparisons and a statistical significance level set at *P* < .05. Stratification of samples into the < 30 ng/mL and ≥ 30 ng/mL strata were based on whether the median anti-FXa value between the 3 instrument-reagent platforms was < 30 ng/mL or ≥ 30 ng/mL.

## 3 Results

**Figure 1:**
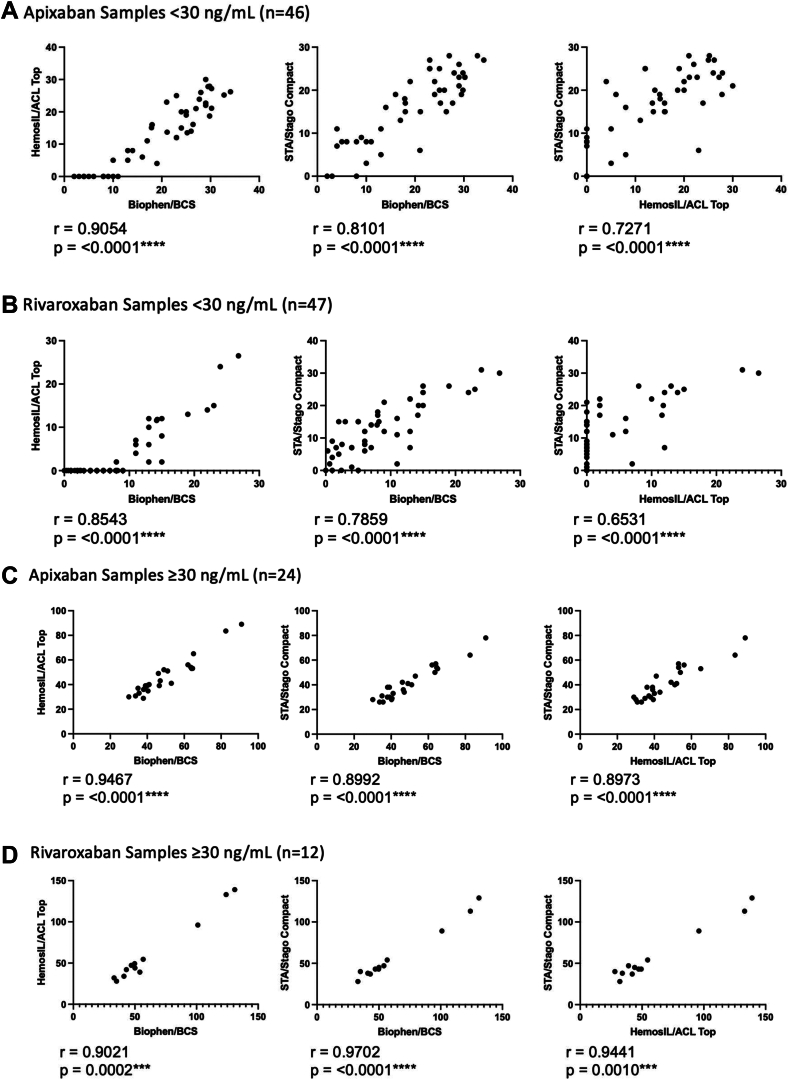
Individual correlation plots between all instrument-reagent combinations at < 30 ng/mL anti-factor Xa (FXa) levels (A, B) and ≥ 30 ng/mL anti-FXa levels (C, D) for both apixaban and rivaroxaban. *P*<0.001∗∗∗_,_*P*<0.0001∗∗∗∗.

Of the 129 unique patient samples tested, 70 were on apixaban and 59 on rivaroxaban. Baseline characteristics between the patients on apixaban and rivaroxaban are presented in Table. A total of 8 patients were removed either due to insufficient sample volume (*n* = 2) or sample mislabeling (*n* = 6). The majority of patients included in our study were in the low-risk surgery group and receiving standard dose DOAC regimens. For both rivaroxaban and apixaban, there was an overall moderate-to-very strong correlation between the anti-FXa levels from each of the instrument-reagent combinations. However, the correlation was weaker in the < 30 ng/mL group (apixaban: *r* = 0.7271-0.9054 ; rivaroxaban: *r* = 0.6531-0.8543) compared with the ≥ 30 ng/mL group (apixaban: *r* = 0.8973-0.9467; rivaroxaban: *r* = 0.9021-0.9702) (Figure 1). Anti-FXa levels determined using the Biophen/BCS and HemosIL/ACL Top combinations showed the strongest correlation in the apixaban < 30 ng/mL (*r* = 0.9054), apixaban ≥ 30 ng/mL (*r* = 0.9467), and the rivaroxaban < 30 ng/mL (*r* = 0.8543) groups. In the rivaroxaban ≥ 30 ng/mL group, anti-FXa levels using the Biophen/BCS and STA/Stago Compact combinations showed the strongest correlation (*r* = 0.9702) (Figure 1).

**Table:** Baseline characteristics of included patients.

Variable	All Patients (*n* = 129)	Apixaban (*n* = 70)	Rivaroxaban (*n* = 59)
Age (y)	75 (68-81)	76 (69-82)	74 (67-80)
Sex			
*Male*	72 (56)	41 (59)	31 (53)
*Female*	57 (44)	29 (41)	28 (47)
Race/Ethnicity			
*White*	125 (97)	70 (100)	55 (93)
*Non-White*	4 (3)	0 (0)	4 (7)
Serum Creatinine(μmol/L)	88 (74-107)	93 (77-110)	82 (72-99)
Creatinine Clearance(mL/min)	69 (51-86)	60 (49-85)	74 (56-87)
Surgical Risk Category			
*High Risk*	28 (22)	12 (17)	16 (27)
*Low Risk*	101 (78)	58 (83)	43 (73)
DOAC Dosing			
*Standard Dose*	100 (78)	52 (74)	48 (81)
*Low Dose*	29 (22)	18 (26)	11 (19)

Data presented as median (IQR) or *n*-value (%).

DOAC, direct oral anticoagulant.

**Figure 2:**
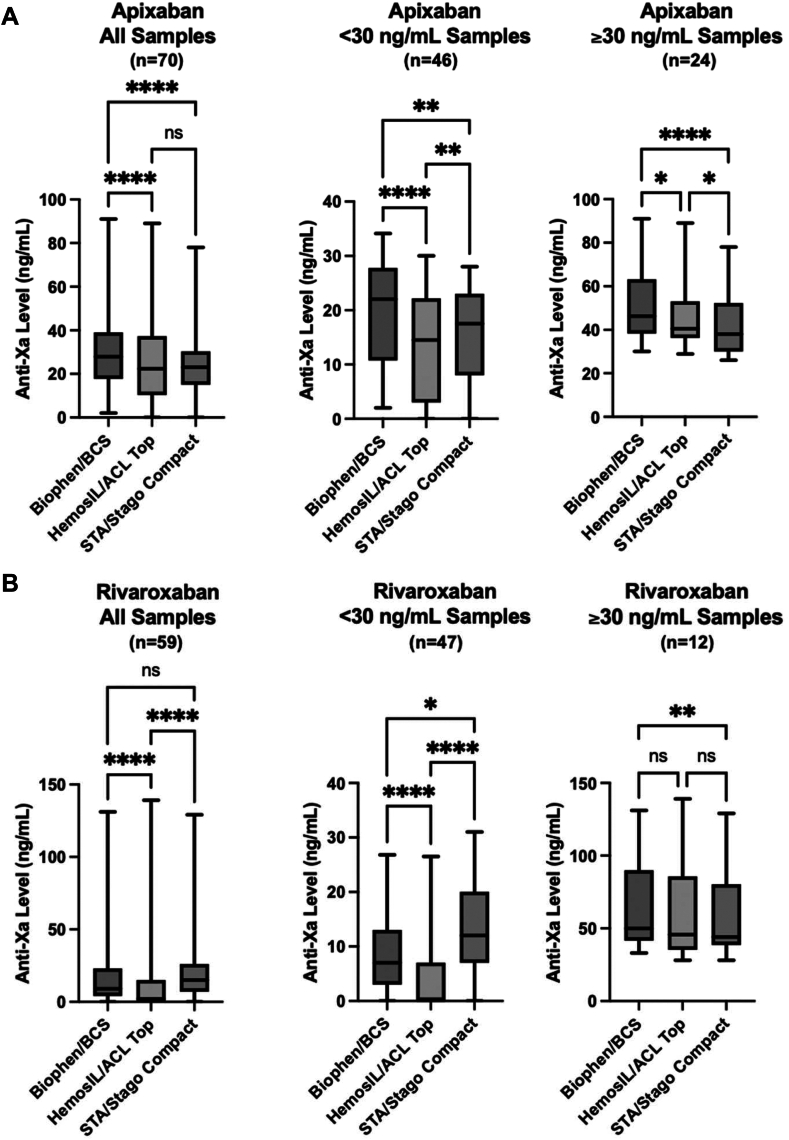
Anti-factor Xa (FXa) activity determined by 3 instrument-reagent combinations and stratified by < 30 ng/mL or ≥ 30 ng/mL cutoffs for patients on either apixaban (A) or rivaroxaban (B). *P*<0.05∗*, P<*0.01∗∗*, P<*0.001∗∗∗*, P<*0.0001∗∗∗∗.

When examining patients on apixaban, there was a significant difference in anti-FXa levels between all instrument-reagent combinations for both the < 30 ng/mL and ≥ 30 ng/mL groups. However, when including all samples, the statistically significant difference was only when comparing Biophen/BCS to both HemosIL/ACL Top and STA/Stago Compact (Figure 2A). When examining patients on rivaroxaban, there was also a significant difference in anti-FXa levels between all instrument-reagent combinations in the < 30 ng/mL group. But for patients in the ≥ 30 ng/mL group, there was only a statistical difference when comparing Biophen/BCS to STA/Stago Compact. Additionally, when examining the entire cohort of patients on rivaroxaban as a whole, the difference was only between HemosIL/ACL Top compared with both Biophen/BCS and STA/Stago Compact (Figure 2B).

**Figure 3:**
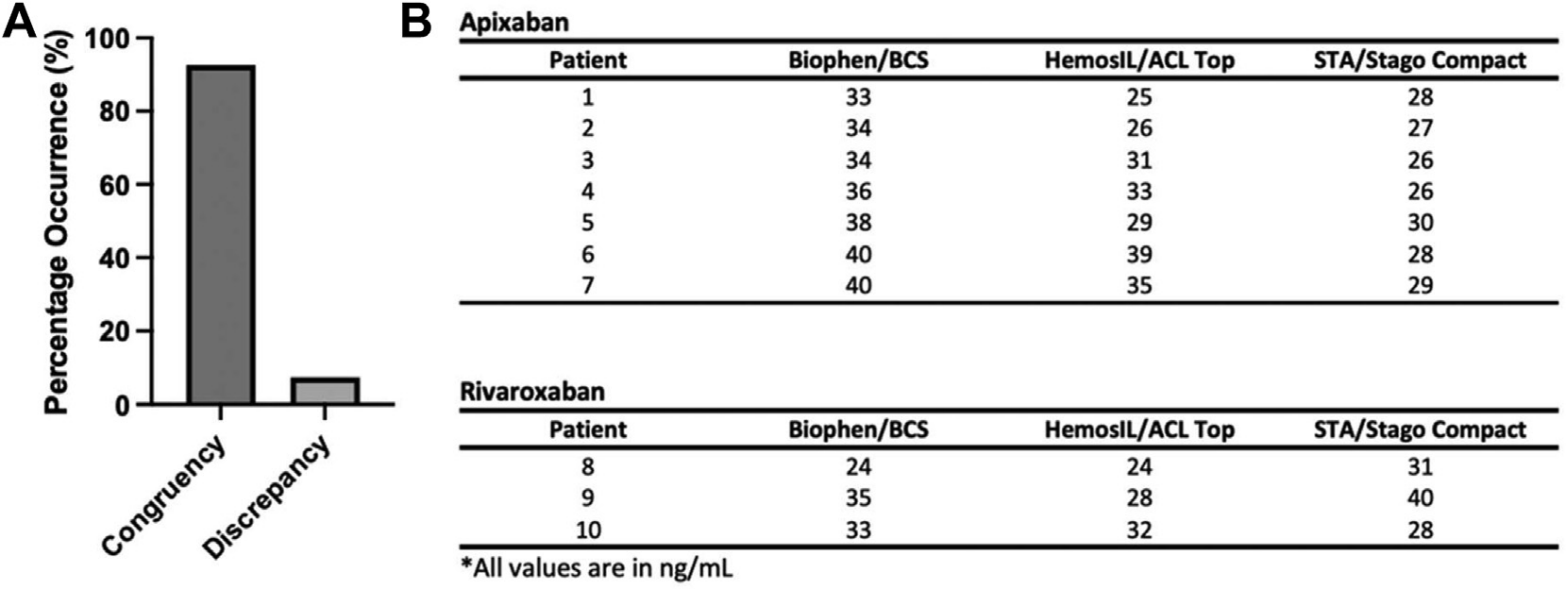
Patients showing discrepant classification across the 30 ng/mL threshold based on the instrument-reagent combination (A). Individual patient plasma anti-factor Xa (FXa) levels that were discrepant across the 30 ng/mL threshold (B).

In 10 of 129 (7.8%) samples, anti-FXa levels were discrepant across the 30 ng/mL threshold; ie, classified as either < 30 ng/mL or ≥ 30 ng/mL depending on the instrument-reagent combination used to determine the anti-FXa level (Figure 3A). Individual anti-FXa results for the 7 apixaban samples and 3 rivaroxaban samples that were classified discrepantly across the 30 ng/mL threshold by the anti-FXa instrument-reagent combinations are shown in Figure 3B. The largest difference between the lowest and the highest anti-FXa value for a patient was 12 ng/mL for either anticoagulant. This difference was seen in a patient taking rivaroxaban with their anti-FXa levels determined on HemosIL/ACL TOP (28 ng/mL) compared with STA/Stago Compact (40 ng/mL).

## 4 Discussion

Our study found a moderate-to-very strong correlation between all 3 instrument-reagent combinations for both apixaban and rivaroxaban. Although there was statistically significant interassay variability in median anti-FXa levels for both drugs, this variability was not clinically significant. This is because at the 30 ng/mL anti-FXa threshold which is commonly used to assess periprocedural bleeding risk, the analyzers discrepantly classified patient samples across this cutoff in only 7.8% of cases. In comparison to other commonly used coagulation tests such as prothrombin time, the Clinical Laboratory Improvement Amendments (CLIA) guidelines accepts a total allowable error of ≤ 15%, also supporting the notion that the errors in classification in our study are within expectations for a clinical coagulation test [27]. The largest difference between analyzers was an anti-FXa level of 12 ng/mL. In addition to this, when there was a discrepancy in the classification between analyzers at this threshold, it was never large enough to cross any other clinically important thresholds such as 50 ng/mL, which has been proposed as a level to consider anticoagulant antidote administration in the setting of severe bleeding [10].

DOACs are increasingly being utilized in clinical practice as a result of their safety and ease of use, with no requirement for routine monitoring. However, in the setting of urgent surgery, procedures or concurrent administration of tissue plasminogen activator, being able to measure the plasma activity of DOACs can help with risk stratifying patients prior to these interventions. Unfortunately, global coagulation tests such as PT and aPTT are unable to accurately predict DOAC levels. Alternatively, anti-FXa levels can be used for helping to risk-stratify patients. While definitive thresholds continue to be studied, 30 ng/mL is currently becoming a threshold of safety in the setting of high-risk surgery [[7], [8], [9], [10]]. Some studies have also proposed 50 ng/mL as an alternative threshold, but in the current literature there is a lack of convincing evidence to support its use specific to high-risk surgery. Boissier et al. [28], describe the distinction between these 2 thresholds and give the opinion that 30 ng/mL should be used for high-risk surgery, while 50 ng/mL could be considered as a threshold for ischemic stroke intravenous thrombolysis. In addition to this, both the French Working Group on perioperative hemostasis and the Scientific and Standardization Committee of the International Society on Thrombosis and Haemostasis, recommend 30 ng/mL as the safety threshold for invasive surgeries with a high risk of bleeding, with the latter proposing 50 ng/mL as the threshold for warranting antidote administration in severe bleeding rather than high-risk surgery [8,10]. We chose to focus on high-risk bleeding, and also, the intention of this study was to focus on interassay variability at low concentrations, and thus we opted to use 30 ng/mL as our target. With previous studies demonstrating interassay variability at these lower anti-FXa levels and with multiple anti-FXa instrument-reagent platforms being used in clinical practice, a critical first step is to understand the inconsistencies in results between these platforms to guide the development of these safety thresholds. Therefore, our study sought to examine the interassay variability among 3 commonly used instrument-reagent platforms while also limiting confounding variables that can affect interassay variability such as using lyophilized samples, DOAC-spiked samples and interlaboratory or interoperator variability.

When compared with previous studies on interassay variability in anti-FXa activity, we found similar levels of variation. For example, Miklič et al. [21], examined the difference between 2 chromogenic anti-FXa assays compared with liquid chromatography with tandem mass spectrometry and found that when looking at samples with anti-FXa levels ranging from approximately 2 to 600 ng/mL the correlation was *r* = 0.98 to 0.99 but dropped to *r* = 0.88 to 0.90 when only examining samples with anti-FXa levels < 50 ng/mL [21]. While our study shows slightly lower correlations overall, this can likely be explained by the fact that we had comparatively lower anti-FXa levels in our samples and then we further stratified these to the category of anti-FXa activity < 30 ng/mL rather than < 50 ng/mL as was done in their study. Similar results were shown in EQA data, which demonstrated increasing variability at lower anti-FXa activity levels [12]. This once again supports the premise that interassay variation is increased at lower anti-FXa levels.

Our study sought to limit confounding factors and the importance of this can be displayed through the results of Asmis et al. [29]. They examined anti-FXa levels in DOAC-spiked plasma samples across 6 different laboratories. For the lowest DOAC concentrations used in this study (71.4-101.3 ng/mL) the accuracy of the anti-FXa levels, compared to high-performance liquid chromatography with tandem mass spectrometry varied from 5.3% to 14.6% and precision from 2.8% and 11.8%. This highlights the potential impact on anti-FXa results that can occur from using DOAC-spiked samples and/or interlaboratory variation.

While previous studies have demonstrated the occurrence of interassay variability in anti-FXa measurements, a strength of this study was that it attempted to control for major confounding laboratory-based factors. These included using nonlyophilized, nonspiked samples, which were analyzed at the same laboratory in parallel to prevent repeated freeze-thaw cycles or requirements of transportation between centers. Not only this, but all samples were also run by a single operator to limit interoperator variability. Therefore, this allowed us to be able to truly determine the level of interassay variability that is due to these commonly used instrument-reagent platforms rather than other factors and help to better identify variations that can be seen in clinical practice. By also determining the frequency of discrepant classification across the 30 ng/mL threshold this study allowed us to better understand the clinical implications of variations seen and how interassay variations could impact clinical decision-making.

A potential limitation of our study was that we chose to include only a portion of the total samples that were collected from patients in the original PAUSE trial, rather than testing all 2624 samples. However, to limit bias related to this we randomly selected the initial 100 patients whom we included in this study. We then enriched our sample by including all patients who were previously known from the original PAUSE study to have anti-FXa levels between 30 and 50 ng/mL with a sufficient volume of frozen plasma to complete testing on all 3 instrument-reagent combinations. Although we did not assay residual DOAC concentration using the gold standard methodology (mass spectrometry), the calibrators for the apixaban and rivaroxaban assays by commercial vendors are known to be accurate, as the calibrator values are established using mass spectrometry. Since a major focus of this study was to determine the clinical implications that could result from interassay variability in commonly used anti-FXa assays, we compared only 3 common instrument-reagent platforms that are currently in use in the majority of clinical laboratories.

## 5 Conclusion

Our study demonstrates that when controlling for factors that introduce laboratory-based variability, there are moderate-to-very strong correlations between 3 commonly used instrument-reagent platforms both above and below the cutoff of 30 ng/mL. Therefore, it is unlikely that any of these differences would lead to clinically important changes in patient management.

As anti-FXa levels are currently being analyzed in hospital laboratories on multiple different instrument-reagent platforms, a critical first step toward improving research on how to interpret anti-FXa levels is to understand how these values from differing platforms can be compared with each other. Our study improves this understanding and as such will aid in the development of high-risk thresholds and assessment of periprocedural bleeding risk in the future.
