# Supplementary material for: Investigating interassay variability between direct oral anticoagulant calibrated anti–factor Xa assays: a substudy of the perioperative anticoagulation use for surgery evaluation (PAUSE) trial
Source: Res Pract Thromb Haemost. 2025 May 23;9(4):102899. doi: 10.1016/j.rpth.2025.102899 (PMC12240172; doi:10.1016/j.rpth.2025.102899)
Supplement: Supplementary Material [file mmc1.docx]

**Declaration of Interest Statement**

The authors declare that they have no known competing financial interests or personal relationships that could have appeared to influence the work reported in this paper.

The author is an Editorial Board Member/Editor-in-Chief/Associate Editor/Guest Editor for this journal and was not involved in the editorial review or the decision to publish this article.

The authors declare the following financial interests/personal relationships which may be considered as potential competing interests:

RB has no conflicts of interest to disclose.

RS has no conflicts of interest to disclose.

KAM has no conflicts of interest to disclose.

MS has no conflicts of interest to disclose.

AS has received research support from Boehringer Ingelheim and Janssen, and consulting fees from Janssen, Bristol-Meyer Squibb/Pfizer Alliance, Sanofi, Astra Zeneca, Boehringer Ingelheim, Bayer, and is a member of the ATLAS group.

SS Honoraria for serving on data safety monitoring boards for Bayer, Boehringer-Ingelheim, Moderna, Regeneron and Sanofi; on a steering committee for Octapharma;  on event adjudication committee for Takeda.  Research grant to my institution from Octapharma

JD has received, in the past 3 years, consulting and/or lecture fees from Pfizer, Leo Pharma, Fresinius Kabi.
