# Supplementary material for: Investigating interassay variability between direct oral anticoagulant calibrated anti–factor Xa assays: a substudy of the perioperative anticoagulation use for surgery evaluation (PAUSE) trial
Source: Res Pract Thromb Haemost. 2025 May 23;9(4):102899. doi: 10.1016/j.rpth.2025.102899 (PMC12240172; doi:10.1016/j.rpth.2025.102899)
Supplement: Supplementary Data [file mmc2.docx]

**Technical Comment Responses**

**Manuscript Number: RPTH-25-00113
Investigating inter-assay variability between DOAC calibrated anti-factor Xa assays: A substudy of the PAUSE trial.**

The following technical comments were discussed upon initial submission of the revised manuscript and will be addressed here:

**1- Please provide four bullet points under the heading ‘Essentials’. The bullet points should be categorized as follows: one for background, one for setting and two for results/significance/conclusion (please do not include these subheadings). This section should immediately precede the abstract. Please ensure that these points are written in lay language and are no more than 98 characters each, including spaces. If abbreviations are used, please write the term in full with the abbreviation in brackets in the first instance, for example, ‘deep vein thrombosis (DVT)’.**

*Response:* We have now provided an ‘Essentials’ component to our revised manuscript as requested.  **2- Please remove the figures from the clean manuscript and upload them separately, preferably in EPS or TIFF format. Figure legends may remain in the manuscript file.**

*Response:* Thank you for this comment, we have now uploaded an additional file titled “Manuscript Tables and Figures” to address this comment. **3- To provide fuller information on the socio-cultural determinants of health of the studied population, research involving human subjects should include race/ethnicity of participants in Table 1. If this is not available, discuss how it might be a limitation not to have this information.**

*Response:* We thank the reviewers for highlighting this, and as such we have now updated our Table 1 to include race/ethnicity data.  **4- Currently, the corresponding author information does not match between the title page of the manuscript and the submission metadata: Rita Selby (Title page) vs. Ryan Baker (Metadata). Please confirm the preferred corresponding author listing for your manuscript.**

*Response*: Thank you for highlighting this discrepancy. We would like to have Rita Selby as the official corresponding author for the publication as shown in the title page. Author Ryan Baker has been completing the online submissions of the manuscript to RPTH, but does not wish to be the corresponding author for the published manuscript. Please reach out to us if this causes a conflict and we would be happy to address any concerns.

**5- We noticed that two identical clean versions of the manuscript have been uploaded. Could you please remove one of the duplicate clean files. We only require one clean version and one red-lined version for review.**

*Response:* Thank you for clarifying this, we have now uploaded one clean version and one red-lined version for review which are titled “Anti-Factor XaDOAC-PAUSESubStudy-2ndRevisionCLEAN” and “Anti-Factor XaDOAC-PAUSESubStudy-Submit_TrackChanges”, respectively.
